# Case Report: Efficacy of albumin dialysis for the reversal of bile cast nephropathy-induced acute kidney injury

**DOI:** 10.3389/fneph.2023.1256672

**Published:** 2023-10-10

**Authors:** Aaron G. Issac, Michael A. Yu, Desiree M. Rogers, Ram M. Subramanian

**Affiliations:** ^1^ Department of Medicine, Emory University School of Medicine, Atlanta, GA, United States; ^2^ Division of Digestive Diseases, Emory University School of Medicine, Atlanta, GA, United States; ^3^ Division of Critical Care Medicine, Emory University School of Medicine, Atlanta, GA, United States

**Keywords:** bile cast nephropathy, molecular adsorbent recirculating system, extracorporeal liver support, acute kidney injury, case report

## Abstract

**Background:**

Bile cast nephropathy (BCN) is an underdiagnosed renal complication associated with severe hyperbilirubinemia and is seen in patients with liver failure who have cholestatic complications. BCN-induced acute kidney injury (AKI) can require hemodialysis (HD), and the molecular adsorbent recirculating system (MARS) is a potentially useful therapeutic option.

**Case summary:**

A 57-year-old male presented with jaundice persisting for 1 month, with laboratory test results indicative of hyperbilirubinemia and AKI. Abdominal imaging and a biopsy confirmed biliary ductal dilation secondary to a pancreatic head mass. The patient had rapidly progressive renal failure and refractory hyperbilirubinemia, despite biliary decompression, and was started on HD. Subsequent therapy with albumin dialysis therapy using MARS was successful in reversing the AKI, the cessation of HD, and the restoration of native renal function.

**Conclusion:**

In the setting of BCN-induced AKI, timely initiation of MARS can provide a useful therapeutic strategy to reverse renal dysfunction and facilitate intrinsic renal recovery.

## Introduction

Bile cast nephropathy (BCN) is a rare and potentially life-threatening renal complication associated with severe hyperbilirubinemia, particularly in patients with cholestatic conditions or those experiencing liver failure (1–3). It is characterized by the formation of bile casts within the renal tubules, leading to acute kidney injury (AKI) and, in severe cases, the need for renal replacement therapy. The pathogenesis of BCN is not yet fully understood, but it is believed to involve the precipitation of bile acids, bilirubin, and other bile components within the renal tubules, causing tubular obstruction, inflammation, and oxidative stress (2, 3). Typically a diagnosis of exclusion, and one confirmed by renal biopsy, BCN is felt to be underdiagnosed. A recent study retrospectively evaluating renal biopsies found that BCN was present in almost 45%–72% of autopsies of patients with cirrhosis (4, 5).

The management of BCN presents a clinical challenge as current therapies primarily focus on addressing the underlying source of cholestasis and providing supportive care for the associated renal dysfunction. Treatment strategies include optimizing fluid balance, ensuring appropriate electrolyte management, and addressing the precipitating factors of liver dysfunction, such as obstructive jaundice, infection, or drug toxicity (2, 3). In some cases, renal replacement therapy such as hemodialysis (HD) may be necessary to manage severe AKI secondary to BCN. In refractory cases, extracorporeal liver support devices utilizing albumin dialysis may help to reverse AKI (6, 7).

**Figure 1:**
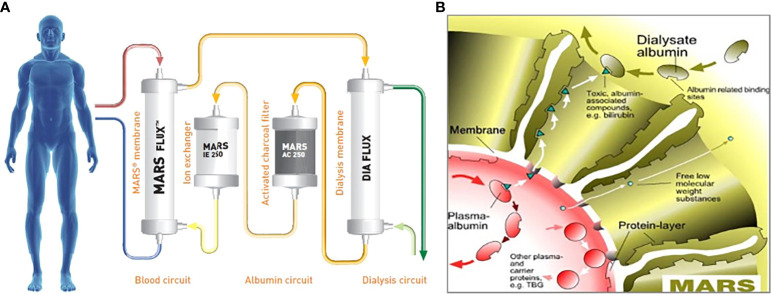
**(A)** Details of the molecular adsorbent recirculating system (MARS) circuit, which involves a dialysate of albumin solution passing through a MARS dialysis membrane and then subsequently through a CRRT membrane, charcoal filter, and anion exchanger. **(B)** Diagram of the MARS membrane, which illustrates the albumin dialysate, and the passage of both water-soluble low-molecular-weight and larger albumin-bound toxins across the membrane. CCRT, continuous renal replacement therapy; TBG, thyroxine-binding globulin. Reproduced with permission from Baxter.

The molecular adsorbent recirculating system (MARS) is an extracorporeal liver support system that has shown promise in the treatment of various liver diseases, including acute liver failure (ALF), acute-on-chronic liver failure as a bridge to transplant, and intractable pruritus (8–10). MARS functions by removing albumin-bound toxins, including bilirubin and bile acids, from the patient’s blood through a combination of continuous renal replacement therapy (CRRT) and the addition of adsorption therapy via the MARS circuit (11, 12) (**Figure 1**). With regard to BCN-associated AKI, the strategy of using MARS aims to reduce the burden of cholestasis to mitigate the toxic effects of bilirubin on the renal tubules and potentially promote renal recovery. The application of MARS in the treatment of BCN has been reported in a few case reports with promising results (6, 7).

In this case report, we describe a patient with obstructive jaundice and BCN requiring HD who was successfully treated using MARS. We will discuss the presentation, diagnosis, and management of the patient’s BCN and the potential benefits of MARS therapy for patients with similar clinical profiles. Furthermore, we will explore the current literature on the utility of MARS for treating BCN and other liver-related renal complications, highlighting the potential for this innovative therapy to improve patient outcomes and reduce the need for renal replacement therapy.

## Case description

**Figure 2:**
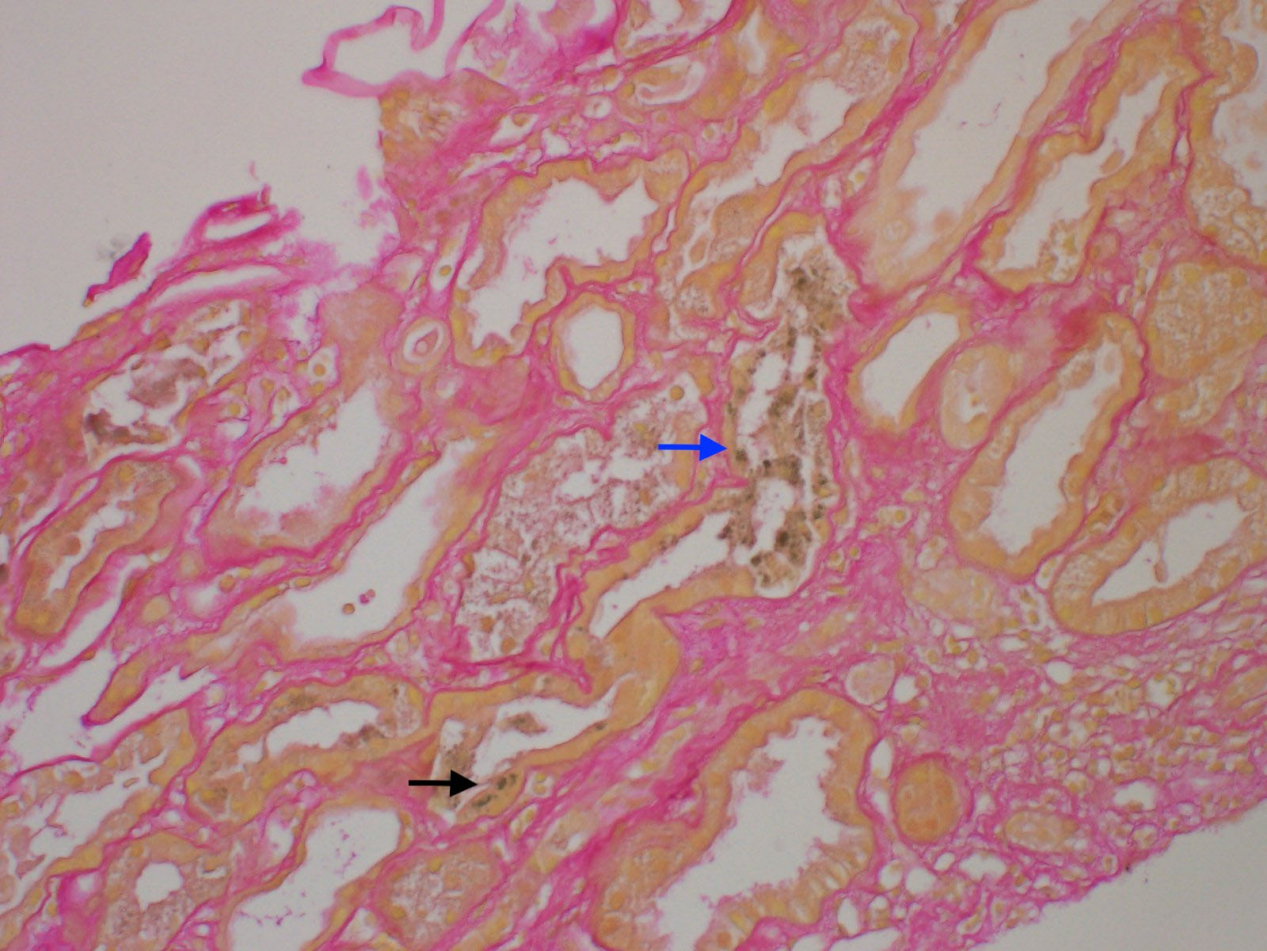
Light microscopy of renal biopsies stained with hematoxylin and eosin and showing bile casts with sclerotic glomeruli. Mild interstitial fibrosis and tubular atrophy are present, involving 10% of the cortical surface (grade 1 IFTA). Intratubular green-tinged casts (blue arrow) are present. Green pigment is also seen within the proximal tubular epithelium cytoplasm (black arrow). IFTA, interstitial fibrosis and tubular atrophy.

A 57-year-old male with diffuse large B-cell lymphoma who had been treated with chemotherapy, but was in remission for over 10 years, presented with jaundice persisting for 1 month, fatigue, dark urine, and pruritus. On admission, the patient had scleral icterus, with laboratory test results notable for a total bilirubin level of 22.3 mg/dL (381.3 μmol/L) and a serum creatinine level of 2.0 mg/dL (176.8 μmol/L) with an unclear baseline. An MRI scan showed biliary ductal dilation and signal abnormality of the pancreatic head threatening malignancy. Subsequent endoscopic retrograde cholangiopancreatogram (ERCP) on hospital day 6 demonstrated a high-grade stricture at the distal common bile duct threatening external pancreatic mass compression with a subsequent unsuccessful biliary stent placement for decompression. Brushings were used as diagnostics for pancreatic adenocarcinoma. On hospital day 7, an internal/external biliary drain for biliary decompression was successfully placed via interventional radiology. Despite this, the patient had persistent hyperbilirubinemia that initially improved to 13.2 mg/dL (225.7 μmol/L) before increasing back to 21.9 mg/dL (374.5 μmol/L). The patient also had worsening renal function up to a maximum creatinine level of 6.3 mg/dL (556.9 μmol/L), with oliguria progressing to anuric renal failure requiring the initiation of high-flux HD with conventional dialysate on day 14. The workup to investigate secondary causes of liver and renal dysfunction, including serum and urine protein electrophoresis, viral and autoimmune hepatitis laboratory tests, urinalysis and culture, 24-h urine protein, bone marrow biopsy, and blood cultures, was unrevealing. A renal biopsy demonstrated acute tubular necrosis secondary to BCN (**Figure 2**).

**Figure 3:**
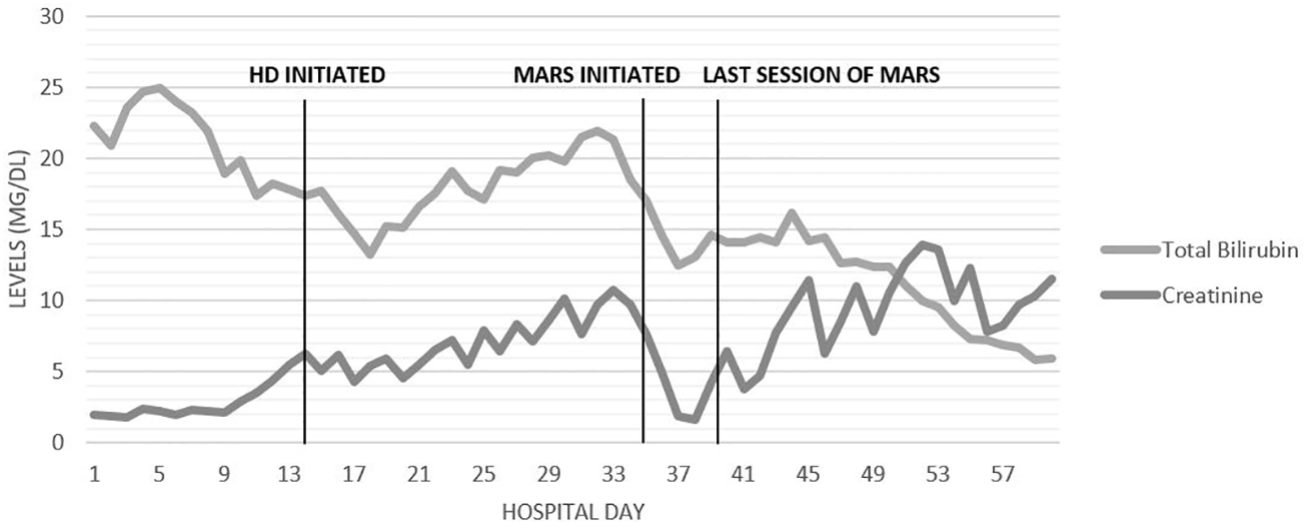
Timeline of days in the hospital and trends in the levels of creatinine and total bilirubin (mg/dL).

Given these findings, the patient was treated with five sessions of MARS therapy. **Figure 3** shows the total bilirubin levels decreasing after the MARS therapy. On hospital day 60, the patient was discharged with a total bilirubin level of 5.9 mg/dL (100.9 μmol/L). The patient still required HD at discharge. One month after discharge, the patient’s urine output had increased, and his creatinine level had stabilized without HD at 1.5 mg/dL (132.6 μmol/L), and HD was discontinued. With the resolution of his hyperbilirubinemia and renal failure, he was able to commence chemotherapy and radiation without further delay, and he achieved a good clinical response with minimal side effects. At the 9-month follow-up he had maintained an Eastern Cooperative Oncology Group (ECOG) performance status score of 0 to 1 but was found to have small metastatic lesions within the liver and so was restarted on chemotherapy.

## Discussion

Initially introduced in 1993 by two German nephrologists, Mitzner and Stange, MARS is an extracorporeal liver support system with applications in the treatment of various liver diseases, including ALF, acute-on-chronic liver failure as a bridge to transplant, and intractable pruritus, as well as drug toxicities (8–10, 12). MARS gained accepted clinical use in the late 1990s. MARS works owing to the blood flowing countercurrently to an albumin dialysate solution that absorbs albumin-bound toxins. The albumin is then regenerated as it passes through activated charcoal and an anion exchange resin, while water-soluble toxins are cleared using a conventional dialysis solution (13). Other extracorporeal liver support devices exist, such as Prometheus^®^ and single-pass albumin dialysis (SCAD). Prometheus uses fractioned plasma separation and adsorption in which a conventional dialysis solution is used, although the patient’s blood directly passes through an albumin-permeable filter when albumin-bound toxins are cleared (14, 15). SCAD uses a 4.4% albumin solution, versus a 20% albumin solution in MARS, as a direct dialysate solution in addition to a conventional dialysate solution to remove both water and albumin-bound toxins (16).

In this case report, we present a patient with BCN who was successfully treated using MARS in a critical care setting. Patients with ALF may present similarly with symptoms related to hyperbilirubinemia. MARS and other forms of extracorporeal albumin dialysis may be considered in ALF as a bridge to liver transplant (17). A multicenter randomized controlled trial of MARS in patients with ALF listed for emergency liver transplant showed no clear survival benefit of MARS therapy (*n* = 54) at 6 months and 1 year when compared with standard medical therapy (*n* = 49). However, in the secondary analysis, patients who received three or more sessions of MARS had significantly improved survival rates compared with patients who received fewer than three treatment sessions, suggesting that three or more sessions may be beneficial in patients with ALF (18, 19). More recently, a retrospective cohort study that compared patients with ALF who received MARS (*n* = 104) with propensity score-matched controls undergoing standard medical therapy (*n* = 416) found MARS to be associated with improved 21-day transplant-free survival rates, biochemical variables, and hemodynamics (9).

The early use of MARS in the management of BCN appears to be a promising therapeutic approach, as demonstrated by our patient’s clinical improvement and renal recovery. Albumin dialysis is effective in reducing bilirubin levels and likely sequelae of high bilirubin burdens, as well as in patients with hepatic encephalopathy (17, 20). By removing albumin-bound toxins, including bile acids and bilirubin, from the patient’s blood through a combination of dialysis and adsorption, MARS is believed to remove toxin burdens to help promote renal recovery in cases of BCN-associated AKI.

Our patient’s hyperbilirubinemia did initially improve slightly on biliary decompression with placement of an internal/external biliary drain on hospital day 7, but it began to increase again on day 17 to day 33 (**Figure 3**). This is potentially explained by the impaired bile acid excretion in the setting of renal failure from BCN, in addition to hepatic synthetic dysfunction from hyperbilirubinemia. Although bilirubin levels did improve, levels plateaued as intermittent hemodialysis does not eliminate albumin-bound toxins such as serum bilirubin (21). MARS therapy was considered and ultimately started to reverse the severe hyperbilirubinemia. We believe that MARS was critical in mitigating the burden of hyperbilirubinemia exacerbating the severity of BCN, thereby facilitating subsequent renal recovery. The ongoing reduction in hyperbilirubinemia following the MARS therapy was likely from improvements in both renal and hepatic function.

Although multiple cases of BCN-associated AKI have been reported in the literature, it is likely underdiagnosed because BCN-associated AKI requires a renal biopsy for confirmation (4, 5). No current therapeutic guidelines exist for the treatment of BCN-associated AKI. Initial interventions, such as relieving biliary obstruction via ERCP with stent placement and HD, can be useful especially if implemented early on, although it is unclear how this affects the natural disease course (1). MARS has been used as a treatment strategy in one other case in our literature search for BCN-associated AKIs, although another case of cholestasis leading to renal dysfunction improved with MARS therapy (**Table 1**) (6, 7). This strategy aims to reduce the burden of cholestasis in the hope of minimizing and eliminating the toxic effects of bilirubin on the renal tubules. This is similar to a more common indication for MARS therapy, refractory pruritus, in which patients with severe hyperbilirubinemia experience decreasing bilirubin levels and improvement of their pruritus (8). Given the renal recovery in our patient, patients with renal failure with suspected or proven BCN should be considered early in their disease course for albumin dialysis therapy using MARS.

**Table 1:** Literature review of cases of cholestasis leading to renal dysfunction treated with extracorporeal liver support devices.

First author, year of publication	Patient age (years), sex	Etiology	Total bilirubin peak level (mg/dL)	Creatinine peak level (mg/dL)	Treatment	Outcome
Sens et al., 2016	37, male	*TCF2* mutation	28	5.88	Eight SCAD and one MARS sessions	Liver and kidney transplant
Saich et al., 2005	34, male	Benign recurrent intrahepatic cholestasis (BRIC)	Unknown	2.11	Three MARS sessions	Liver and kidney function returned to baseline

MARS, molecular adsorbent recirculating system; SCAD, single-pass albumin dialysis; TCF2, transcription factor 2.

## Data availability statement

The original contributions presented in the study are included in the article/supplementary material. Further inquiries can be directed to the corresponding author.

## Ethics statement

This was a case report, and thus, no IRB waiver was required. The patients’ informed consent was obtained. The studies were conducted in accordance with the local legislation and institutional requirements. The participants provided their written informed consent to participate in this study. Written informed consent was obtained from the individual(s) for the publication of any potentially identifiable images or data included in this article.

## Author contributions

AI: Data curation, Writing – original draft, Writing – review & editing. MY: Conceptualization, Writing – original draft, Writing – review & editing. DR: Conceptualization, Writing – review & editing. RS: Conceptualization, Writing – review & editing.
